# DBT-K for Adolescents: Feasibility and Preliminary Outcomes of a Creative Eight-Week, DBT-Based Transdiagnostic Skills Group

**DOI:** 10.3390/children13020172

**Published:** 2026-01-26

**Authors:** Elias Legat, Lucas Rainer, Florian Huber, Belinda Plattner, Andreas Kaiser, Pauline Schaffer, Helena Gampe, Kornelius Winds

**Affiliations:** 1 Department of Child and Adolescent Psychiatry and Psychotherapeutic Medicine, Salzburger Landeskliniken, Paracelsus Medical University Salzburg, Ignaz-Harrer-Straße 79, 5020 Salzburg, Austria; e.legat@salk.at (E.L.); f.huber@salk.at (F.H.); belinda.plattner@teach.pmu.ac.at (B.P.);; 2 Department of Neurology, Neurocritical Care and Neurorehabilitation, Salzburger Landeskliniken, Member of the European Reference Network, EpiCARE, Paracelsus Medical University Salzburg, Ignaz-Harrer-Straße 79, 5020 Salzburg, Austria; 3 Centre for Cognitive Neuroscience Salzburg, University of Salzburg, Ignaz-Harrer-Straße 79, 5020 Salzburg, Austria; 4 Department of Psychiatry, Psychotherapy and Psychosomatics, Salzburger Landeskliniken, Paracelsus Medical University Salzburg, Ignaz-Harrer-Straße 79, 5020 Salzburg, Austria; 5 Department of Educational Science, University of Education Salzburg, Akademiestraße 23-25, 5020 Salzburg, Austria; pauline.schaffer@phsalzburg.at

**Keywords:** dialectical behavior therapy, skills training group, adolescents, expressive arts-based intervention, transdiagnostic

## Abstract

**Highlights:**

**What are the main findings?**

- An eight-week, creative DBT-based transdiagnostic skills group (DBT-K) demonstrated feasibility for highly burdened outpatient adolescents, yielding significant reductions in negative emotions both longitudinally and within-session.
- While negative emotions decreased and the utilization of emotional expression strategies significantly increased, positive emotions and self-esteem remained stable throughout the intervention period.

**What are the implications of the main findings?**

- Creative, brief, DBT-informed group interventions are a potentially viable and accessible treatment option for adolescents with complex comorbidities and problematic internet use in routine clinical care.
- These preliminary findings highlight the importance of targeting core transdiagnostic processes, such as emotion regulation and self-esteem, in intensive, age-appropriate psychiatric settings.

**Abstract:**

**Background**: Mental disorders and emotion dysregulation are highly prevalent in adolescence, yet access to intensive, age-appropriate treatments remains limited. This pilot study examined the feasibility and preliminary effects of a creative, 8-week, DBT-based transdiagnostic skills group for adolescents (DBT-K). **Methods**: 53 outpatients (aged 13–18, 50 female) with heterogeneous psychiatric diagnoses completed baseline self-report measures of internet use, emotion regulation, self-esteem, temperament, and psychopathology. During eight weekly group sessions, positive and negative emotions were assessed before and after each session; self-esteem and emotion regulation strategies were reassessed at the final session. Linear mixed models were used to analyze trajectories of affect and self-esteem. **Results**: Adolescents showed high baseline internalizing symptoms, low adaptive emotion regulation, and low self-esteem, with a substantial proportion meeting criteria for problematic internet use. Across sessions, negative affect exhibited a significant reduction, with a significant main effect of time and pre- vs. post-session condition. Positive emotions showed no systematic pre-post change and a small decline over time. Self-esteem height remained stable across sessions. No significant changes emerged for total adaptive or maladaptive strategies, but expression of emotions increased significantly. **Conclusions**: These findings suggest that a brief, creative DBT-based group is feasible in a highly burdened, transdiagnostic outpatient sample and was associated with a reduction in negative affect and an increase in emotional expression. However, the lack of control group, high attrition, short duration, and absence of follow-up emphasize that conclusions are preliminary.

## 1. Introduction

Adolescence is often portrayed as a period of relative physical health, with lower mortality and morbidity compared to adulthood [1]. Nevertheless, it is also a developmentally sensitive phase in which the majority of mental disorders first emerge. Epidemiological data indicate that more than 70% of mental disorders onset before the age of 25, underscoring adolescence as a high-risk window for the development of psychopathology [2]. In recent years, the prevalence of mental disorders among children and adolescents has become increasingly concerning. In a representative study of Austrian youth (MHAT; Mental Health in Austrian Teenagers), 10–18-year-olds showed a 6-month prevalence of 23.9% and a lifetime prevalence of 35.8% for psychiatric disorders, with anxiety and depressive disorders being most frequent [3]. Even in health care systems with well-developed psychotherapeutic services, many adolescents face delayed access, long waiting lists, and barriers to age-appropriate, evidence-based treatment [4]. This treatment gap has stimulated the development of brief, structured, and scalable psychosocial interventions that can be implemented in routine outpatient care.

One promising approach is to focus on transdiagnostic mechanisms that cut across traditional diagnostic categories, thereby enabling intervention modules to be used in heterogeneous clinical samples [5]. Among these mechanisms, emotion regulation (ER) and self-esteem (SE) have received particular attention in adolescence [6,7]. ER refers to “all the extrinsic and intrinsic processes responsible for monitoring, evaluating, and modifying emotional reactions to accomplish one’s goals” [8]. Healthy children gradually develop a range of strategies for regulating their emotions strategies, which become more differentiated during adolescence [9,10]. However, many adolescents rely heavily on maladaptive strategies such as avoidance, suppression, or rumination, which are linked to internalising and externalising problems [11,12]. Therefore, intervening in the regulation of emotions during adolescence is considered crucial for promoting emotional and social competence later in life as well as reducing the risk of psychopathology in adulthood [13].

Within this process, SE has been proposed as a related transdiagnostic factor [14]. SE reflects an individual’s global evaluation of self-worth, expressed in more positive or negative self-views [15]. During adolescence, SE is closely linked to psychological well-being, social functioning, and negative affect [14,16]. Low SE has been associated with depression, anxiety, and borderline personality disorder [17,18] as well as with less resilient responses to stress and daily challenges [19,20]. Additionally, adolescents with low SE seem to be more likely to engage in problematic internet use (PIU), particularly problematic social media use (SM) [21,22,23,24]. Although research on SE as a transdiagnostic process in youth is still comparatively limited, its involvement in various disorders such as eating disorders and personality disorders suggests that it may be a clinically relevant target for transdiagnostic interventions [7,25,26]. One treatment approach that directly targets both ER and self-related functioning is dialectical behaviour therapy (DBT), a cognitive-behavioural approach. Originally developed for chronically suicidal women meeting the criteria for borderline personality disorder, DBT has since been adapted for use with a broader range of clinical populations [27,28,29]. The standard DBT programme includes individual therapy, telephone coaching when needed, a therapist consultation team, and weekly skills training groups. The Skills training element aims to reduce dysfunctional behaviour and emotional dysregulation by teaching and practising new behavioural skills. More than 60 skills are typically organised into four core modules: mindfulness (MI), emotion regulation (ER), interpersonal effectiveness (IE), and distress tolerance (DT) [28]. DBT was later adapted for adolescents (DBT-A) [30], and evidence suggests comparable effectiveness to adult DBT in reducing self-harm and other high-risk behaviours [31,32]. DBT-A retains most of the original skills modules, complementing them with adaptations specific to adolescents and family components [33,34,35]. DBT-A has been found to be an effective treatment for reducing suicidal ideation and self-harm in adolescents [36].

Beyond disorder-specific protocols, DBT-based skills training groups have increasingly been evaluated as transdiagnostic interventions. In adults, transdiagnostic DBT skills groups have been shown to reduce emotion dysregulation, anxiety, and depressive symptoms, while improving substance use, impulsivity, suicidality, and quality of life, in heterogeneous clinical samples [5,37,38]. For example, Neacsiu et al. [5] demonstrated that a 16-week stand-alone DBT skills group was more effective than an activities-based support group at reducing emotion dysregulation and anxiety. Southward et al. [38] showed that increases in DBT skill use and perceived skill effectiveness were associated with lower daily stress, anxiety, and depression across a 14-week programme. Durpoix et al. [37] showed that a transdiagnostic stand-alone skills group consisting of 16 weekly sessions, followed by a one-year follow-up, improved substance use, impulsivity, suicidal thoughts, emotional instability, and quality of life. Taken together, these studies highlight the potential of DBT skills training to address shared mechanisms such as ER across diagnostic categories.

In children and adolescents, cognitive behavioural therapy (CBT) is the most frequently used approach for transdiagnostic group interventions consisting of core components such as ER, mindfulness (MI), and cognitive restructuring [39]. Studies show reduction in anxiety symptoms and improvements in overall psychological distress as well as emotional disorders in inpatient settings [40,41]. A wide range of transdiagnostic group interventions for children and adolescents in different settings has been developed and evaluated. However, many of these interventions are not reported in a sufficiently rigorous manner, which indicates a risk of bias. In addition, evidence for transdiagnostic interventions in adolescent samples remains limited, particularly in full DBT-A programmes [39]. Only a few studies have directly examined effects on SE, with one trial in male adolescents reporting DBT-related increases in SE, possibly via improvements in ER, IE, and general life skills [42].

Despite growing support for DBT-based interventions, the format of skills training groups may pose challenges in adolescence. Manualised, worksheet-heavy groups can resemble classroom teaching and may be perceived as insufficiently engaging or age-appropriate. To address these barriers, creative and playful elements—such as role-play, storytelling, arts-based tasks, or movement—may help to foster multimodal learning, enhance engagement, and facilitate emotional expression. However, there is currently no empirical data on creative, brief DBT-inspired skills groups for adolescents referred for transdiagnostic treatment.

To address this gap, the present study evaluated an 8-week creative DBT-A–based transdiagnostic skills training group (DBT-K) implemented in an outpatient child and adolescent psychiatry service. The intervention combined core DBT skill modules (MI, DT, ER, IE, and self-related work) with expressive and playful elements. It was offered to adolescents with diverse psychiatric conditions and substantial comorbidity.

This project was conceived as an uncontrolled pilot and feasibility study. Our primary aims were to determine whether a brief, creative DBT-based skills group could be feasibly implemented within routine outpatient services for youth, and to examine preliminary changes in negative (NE) and positive emotions (PE) across sessions. Secondary aims included assessing changes in self-esteem (SE) and emotion regulation (ER) strategies, with particular focus on emotional expression, and exploring associations between affect, ER, SE, and broader clinical features such as psychopathology, temperament, and problematic internet use (PIU). Given DBT’s focus on emotion dysregulation, we anticipated reductions in negative affect across the intervention, whereas changes in PE and emotional expression were expected to be more modest or variable.

## 2. Methods

### 2.1. Participants

This study used a naturalistic, uncontrolled, single-arm pre–post design to evaluate an eight-session outpatient creative DBT-A–based skills training group (DBT-K) delivered in routine clinical care. Information about participation in the skills group was provided to patients by their treating child and adolescent psychiatrists, who were informed about the inclusion and exclusion criteria. The treating physicians recommended participation in the group and provided information about the intervention and its process. Upon agreement to participate, patients received written informational material detailing the content and schedule of the group, and registration was completed either by the adolescents themselves or by their legal guardians. Adherence was emphasized, as frequent absences were expected to interfere with both treatment benefit and group dynamics.

A total of 55 children and adolescents undergoing therapeutic outpatient treatment at the clinic were enrolled in the skills training group between 2022 and 2023. Two participants were excluded from the analyses due to insufficient completion of questionnaires, resulting in a final sample of 53 adolescents (50 female, 94.3%) aged 13–18 years (M = 15.6, SD = 1.36).

As the outpatient DBT-K program was newly established, it was accompanied by a questionnaire-based evaluation to assess and refine the intervention for internal clinical purposes. Participants and their legal guardians were verbally informed during treatment that routine clinical instruments (standard evaluation questionnaires) would be used to monitor therapeutic progress and to evaluate this novel approach. To allow dissemination beyond internal quality assurance, the project was subsequently submitted to the Ethics Committee for approval of publication and was approved. This study, therefore, constitutes a retrospective evaluation of data collected as part of routine clinical documentation and treatment monitoring. Only pseudonymised data were processed to ensure anonymity.

Inclusion criteria were: (1) age between 13 and 18 years, (2) current outpatient treatment at our University Clinic, (3) sufficient German language skills, and (4) adequate group aptitude, particularly the capacity to refrain from impulsive or aggressive behaviour toward others. Exclusion criteria included: (1) acute psychotic symptoms (e.g., acute mania or schizophrenia), (2) acute suicidality, (3) pronounced cognitive impairment preventing adequate processing of intervention content, and (4) insufficient reading or comprehension skills preventing completion of self-report measures. Given the transdiagnostic focus of the intervention, participation was open irrespective of primary psychiatric diagnosis. The age range was selected to ensure developmental homogeneity within the group setting. Cognitive exclusions were defined as levels preventing engagement with mindfulness or radical acceptance concepts, as well as hindering the ability to complete self-report measures. Inclusion and exclusion criteria were assessed by the clinician responsible for treatment.

The study was conducted in accordance with the Declaration of Helsinki and approved by the local ethics committee of the Paracelsus Medical University Salzburg (PMU-EK-2024-0020) on 22 November 2024. As the data collection formed part of the standard clinical routine and the analysis was conducted retrospectively on pseudonymised records, the ethics committee of the Paracelsus Medical University Salzburg reviewed and approved the study protocol without requiring additional written consent forms from participants and their legal guardians.

### 2.2. Procedure

The skills group took place at the outpatient clinic of the University Clinics for Child and Adolescent Psychiatry and Psychotherapeutic Medicine, Christian-Doppler University Hospital, Paracelsus Medical University Salzburg. The group was conducted as a closed and progressive format with approximately eight patients per cycle. Sessions were held in the late afternoon during the school term and in the morning during school holidays. Each skills training cycle consisted of eight consecutive weekly sessions, each lasting approximately 90 Minutes. These skills group cycles were conducted continuously at the clinic, and participation was only possible at the beginning of each new closed cycle (i.e., no rolling admission). The participants were verbally informed that a routine progress evaluation would be carried out via questionnaires, which could be completed voluntarily. Therefore, failure to complete those did not lead to exclusion from the group. Patients with mild developmental deficits were assisted in completing the questionnaires.

The intervention was based on the German DBT-A manual [43] and further adapted by integrating creative, expressive, and playful elements, forming a DBT-based “creative” skills training group (DBT-K). The overarching goal was to maintain the core DBT modules while translating content from a predominantly cognitive, worksheet-based format into a more experiential and interactive setting.

Each group cycle comprised eight weekly sessions (approximately 90 min each), organised into the following modules:

- Session 1—Mindfulness (MI):

The mindfulness module focused on experiential engagement with the five senses. Tactile exploration was facilitated through natural materials (e.g., pinecones, stones, leaves, pussy willow catkins, moss). Olfactory stimuli were presented via essential oils, herbs, and spices. Given that gustatory stimuli can evoke distressing associations in trauma-exposed patients, participants were instead asked to describe the taste of different fruits, while the rest of the group attempted to identify the fruit based on the description. Auditory awareness was trained using recordings of environmental sounds (e.g., forest, stream, rain). Visual perception was addressed through a paired exercise in which participants described an image to a partner, who then attempted to draw the scene based solely on the verbal description. Introduction to the concept of “skills”, their purpose and use, and the importance of staying in the present moment. Exercises targeted different sensory modalities (e.g., listening to sounds such as the sea, city, thunderstorm, touching, smelling, tasting) to cultivate mindful awareness. Further mindfulness practice included Origami and playing Mikado.

- Sessions 2–3—Distress Tolerance (DT):

Session 2 introduced stress and DT using the metaphor of a mountain panorama, where the highest peak represented the individual’s main stressor. Participants used body diagrams to locate where stress is felt physically. To illustrate stress reduction by shifting attentional focus away from distress, the group participated in the cognitive diversion task “Stadt–Land–Fluss.” Working in groups of three, participants were given an initial letter and had to rapidly produce words for a series of categories (city, country, river, animal, personal name, and profession). The group that completed the categories first announced completion, and all answers were subsequently read aloud. Session 3 continued the theme by guiding adolescents through an individual “dream journey” and having them symbolically leave stressors behind an imaginary wall. They were also encouraged to try something new (e.g., listening to opera music, eating with the non-dominant hand) to broaden behavioural repertoires. In addition, a range of positive activities was presented (e.g., meeting friends, spending time outdoors, reading, or taking a bath). Participants were asked to indicate with a thumbs-up or thumbs-down gesture whether they would personally find each activity enjoyable. These activities served to broaden behavioral repertoires and enhance behavioral activation by identifying personally rewarding options.

- Sessions 4–5—Emotion Regulation (ER):

In session 4, participants played a favourite song and reflected on the emotions elicited. The purpose of this exercise was to facilitate emotion labeling and to demonstrate that seemingly contradictory emotions can coexist. To highlight the bodily and visual language of emotions, participants were given cards depicting different emotions and were asked to represent them pantomimically, while the remaining group members guessed the emotion. This was followed by an introduction to basic ER concepts, including the “ABC-Healthy” model. In Session 5, emotions and related belief systems were explored by discussing situations that participants had handled successfully and identifying which emotions had contributed to adaptive coping. Participants were also encouraged to observe their experiences from a “bird’s-eye perspective” in order to gain distance, detect recurrent patterns, and differentiate between emotional reactions and underlying beliefs.

- Session 6—Self-esteem/Dogmas (DO):

This session focused on self-related beliefs (“DO”): how they develop and which beliefs participants hold about themselves. Psychodramatic techniques were used at this stage of the intervention. To facilitate rapid entry into the psychodramatic component, the fairy tale Snow White was selected and read aloud. Participants were then assigned roles and asked to identify the core beliefs attributed to their characters (e.g., the stepmother: “Beauty is the primary value; those who are not beautiful have no worth”). Participants subsequently analyzed each character’s behavior to reflect on the difficulties and conflicts resulting from these beliefs.

- Sessions 7–8—Interpersonal Effectiveness (IE):

In session 7, we introduced the “new path” theme. To consolidate and deepen the psychodramatic work from Session 6, the next session focused on analyzing the fairy tale Sleeping Beauty. This time, participants were instructed to alter the plot by modifying the behavioral paths of the protagonists (e.g., inviting the thirteenth fairy, reflecting on prejudices toward her, identifying her strengths, and departing from predetermined narrative patterns). The revised storyline was then enacted within the group to allow the emotional aspects of these changes to be experienced. This exercise fostered narrative flexibility, perspective-taking, and exploration of alternative behavioral pathways through psychodramatic enactment. Then the concept of change and radical acceptance was discussed. Adolescents created posters about desired changes in the real world (e.g., climate, women’s rights). The concept of radical acceptance was introduced, and participants were guided to formulate personal facts that they feel need to be radically accepted.

Session 8 focused on goal pursuit and becoming the protagonist of one’s own life story. Participants reflected on what role they would play in a film about their life, including genre and plot, and practised the DBT concept of validation. In addition, participants took on the role of a “wish fairy” and were invited to articulate qualities or strengths they would bestow upon fellow group members to support them in attaining their goals. This exercise promoted strength recognition, perspective-taking, and interpersonal validation by identifying personal resources relevant to goal attainment.

All sessions began with a brief MI warm-up using an “MI ball” that contained different prompts or questions. Participants responded to the prompt that their thumb touched when catching the ball.

**Figure 1:**
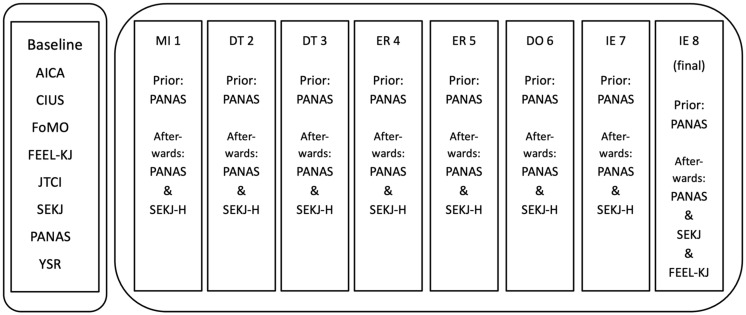
Study’s procedure. Note: MI = Mindfulness, DT = Distress Tolerance, ER = Emotion Regulation, DO = Dogmas, IE = Interpersonal Effectiveness, AICA = Assessment of Internet and Computer Game Addiction, CIUS = Compulsive Internet Use Scale, FoMO = Fear of Missing Out, FEEL-KJ = Questionnaire for the evaluation of emotional regulation in children and adolescents, JTCI = Junior Temperament and Character Inventory, SEKJ = Self-esteem Inventory for Children and Adolescents, PANAS = Positive and Negative Affect Scale, YSR = Youth Self-Report, SEKJ-H = SEKJ height subscale.

Figure 1 shows the assessment procedure: Before the start of the group therapy, participants were asked to fill in a baseline (BL) assessment, which they were asked to submit on the first day of the program. During each session, participants were asked to complete pre- and post-assessments. These tests were suitable for juveniles of that age and consisted of three pages. Each assessment took approximately 3–5 min. To minimize assessment burden and ensure high data quality among the adolescent participants, only brief, validated scales (PANAS, SEKJ-H) were used. As these scales were used consistently, participants became familiar with them, which further prevented survey fatigue within the 90-min session structure. This minimized assessment burden and allowed for quick completion before and after each session. At the end of the final session, three psychometric tests were administered.

### 2.3. Material

Problematic internet use (PIU) was assessed with the Scale for the Assessment of Internet and Computer Game Addiction (AICA) [44] and the Compulsive Internet Use Scale (CIUS) [45], which capture problematic and compulsive use across domains such as gaming, social networking sites (SNS), SM, and streaming. ER strategies were measured with the Questionnaire for the Evaluation of Emotional Regulation in Children and Adolescents (FEEL-KJ) [46], providing adaptive, maladaptive, and “other” strategy scores. Fear of missing out (FoMO) was assessed with the FoMO-scale [47]. Temperament and character traits were captured using the Junior Temperament and Character Inventory (JTCI) [48,49], assessing novelty seeking, harm avoidance, reward dependence, persistence, self-directedness, cooperativeness, and self-transcendence.

Affective states before and after each session were measured with the German version of the Positive and Negative Affect Schedule (PANAS) [50], yielding PE and NE scores. SE was assessed with the Self-Esteem Inventory for Children and Adolescents (SEKJ) [51], including height, stability, and contingency subscales; the height subscale (SEKJ-H) was repeatedly administered across sessions. Psychopathology during the past six months was measured with the Youth Self-Report (YSR) [52], providing internalising, externalising, and total problems scores. When available, T-scores were computed according to the manual, with values between 40 and 60 considered normative; values outside this range were interpreted as sub- or supranormal.

Detailed descriptive statistics for all BL measures are reported in Table S1 in the Supplementary Material.

When available, T-scores provided by the questionnaires’ manuals were calculated. T-scores between 40 and 60 were considered within the normative range; values outside this range were interpreted as sub- or supranormal according to the respective manuals.

### 2.4. Data Analysis

All statistical analyses were conducted using SPSS version 29 [53]. Descriptive statistics were computed for demographics, internet use (AICA, CIUS), ER strategies (FEEL-KJ), FoMO, temperament and character traits (JTCI), PE and NE (PANAS), SE (SEKJ), and internalizing and externalizing symptoms (YSR) at BL. To examine change in affect over the course of the intervention, linear mixed models were calculated separately for PE and NE. Each model included fixed effects for Timepoint (BL plus eight intervention sessions) and Condition (pre vs. post within session), and random intercepts for participants. Due to limited sample size, no interaction term was included. Restricted maximum likelihood (REML) estimation was used to obtain less biased variance estimates. To analyze change in SE height (SEKJ-H) across the intervention, a second linear mixed model was fitted with Timepoint (sessions 1–7) as fixed effect and random intercepts for participants. Changes in ER strategies (FEEL-KJ) from BL to final assessment were analyzed using paired-samples t-tests (or non-parametric equivalents where appropriate) for adaptive, maladaptive, and “other” strategies, as well as total scores. Finally, two-tailed Spearman correlations were calculated to explore associations between internet use–related variables (AICA, CIUS), ER strategies (FEEL-KJ), FoMO, temperament and character traits (JTCI), internalizing and externalizing symptoms (YSR), and PE, NE, and SEKJ-H across the different intervention time points. Given the exploratory nature of this uncontrolled pilot study, all tests were two-tailed with an alpha level of 0.05, and *p* values were not adjusted for multiple comparisons.

#### 2.4.1. Missing Data

Missing data were handled using linear mixed models (LMMs) for the primary analyses of affect and self-esteem trajectories. LMMs are particularly robust for longitudinal clinical data, since they utilize all available data points from each participant and do not require balanced designs. This approach accounts for missing data under the ‘Missing at Random’ (MAR) assumption, which is more suitable for clinical pilot studies than traditional listwise deletion or simple imputation methods. For the pre-post comparisons of emotion regulation strategies (FEEL-KJ), only participants who completed both assessments were included in the pairwise analyses (n = 28); thus, an available case analysis was conducted for these specific measures.

#### 2.4.2. Drop-Out Analysis

Given the observed dropout rate of 53%, additional in-depth analyses were conducted to explore factors associated with adherence and attrition. Feasibility was primarily assessed using adherence indicators, specifically intervention completion status. A binary dropout variable was created using dummy coding, with participants who attended all eight sessions coded as 0 (completion) and those who attended fewer than eight sessions coded as 1 (dropout).

To explore potential factors associated with dropout, participants who completed all sessions were compared with those who did not. Mann–Whitney U tests were used for continuous or ordinal baseline variables (e.g., self-esteem, emotion regulation strategies, internalizing and externalizing symptoms). A comorbidity variable was created using dummy coding, with participants presenting with a single diagnosis coded as 0 (no comorbidity) and those presenting with two or more diagnoses coded as 1 (comorbidity). Associations between categorical variables (comorbidity: yes/no; problematic internet use: yes/no) and dropout were examined using Fisher’s exact test. All feasibility-related analyses were exploratory and intended to inform a more nuanced interpretation of feasibility and acceptability rather than to test confirmatory hypotheses.

## 3. Results

### 3.1. Sample Characteristics and Diagnostic Profile

Psychiatric diagnoses were obtained from routine clinical records. The sample included adolescents with a wide variety of diagnoses from the 10th revision of the International Statistical Classification of Diseases and Related Health Problems (ICD-10) diagnoses (see Table 1). Participants presented with a diverse diagnostic profile. The sample was primarily characterized by anxiety-, stress- or somatoform-related disorders (F40–F48), personality and behavioral disorders (F60–F69), as well as mood disorders (F30–F39), with a high rate of comorbidity where many adolescents met the criteria for at least one additional diagnosis (see Table 1 for detailed distribution).

### 3.2. Baseline Clinical and Transdiagnostic Characteristics

Descriptive statistics for all BL measures are presented in Table S1 in the Supplementary Material. Overall, adolescents reported relatively low levels of problematic computer gaming but higher levels of problematic use of SNS and SM (according to the AICA), with a substantial subset meeting criteria for PIU on the CIUS. Adaptive ER strategies (FEEL-KJ) were generally reduced, with more than half of the sample scoring below the normative range on several adaptive subscales, whereas maladaptive strategies were, on average, within the normative range but at relatively high levels. SE height, stability, and contingency (SEKJ) were all low, with approximately two-thirds of participants scoring below the normative range. In terms of temperament and character (JTCI), adolescents showed elevated harm avoidance and low self-directedness, while other traits were largely within the normative range. BL PANAS scores indicated moderate NE and somewhat lower PE. YSR scores revealed a high internalising symptom burden, with the majority of adolescents above the clinical cut-off and only a minority within the normative range, whereas externalising symptoms were less pronounced.

### 3.3. Course of Positive and Negative Emotions

**Figure 2:**
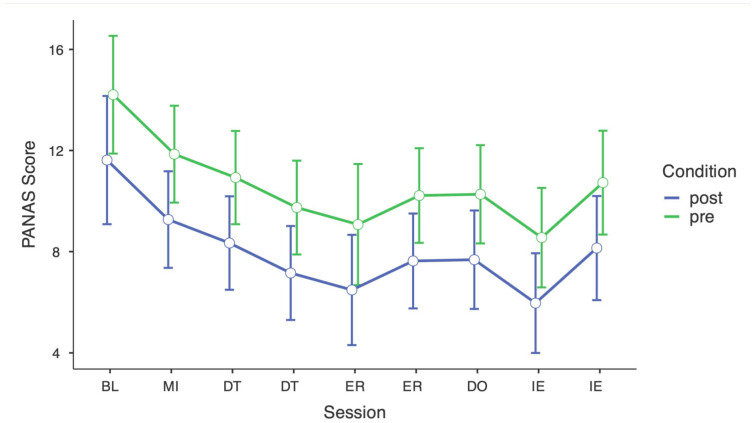
Linear Mixed Model for negative emotions scores across the intervention, including baseline measure. Note: BL = Baseline, MI = Mindfulness, DT = Distress Tolerance, ER = Emotion Regulation, DO = Dogmas, IE = Interpersonal Effectiveness. Error bars represent 95% confidence interval. Pre = pre-intervention, Post = post-intervention.

Linear mixed models were calculated for NE and PE with timepoint and condition (pre vs. post) as fixed effects. For negative affect (NE), the model explained 44.1% of the variance when random effects were included (conditional R^2^ = 0.441), with 7.3% explained by fixed effects (marginal R^2^ = 0.073). There was a significant main effect of timepoint, F(8, 520.8) = 3.32, *p* < 0.001, indicating a gradual decrease in NE from BL through the early sessions, with the largest reduction around the first IE module. NE scores were significantly lower at the final session compared to BL, t(31) = −2.64, *p* = 0.009. Across all sessions, NE was also significantly lower after each session than before, F(1, 512.3) = 25.94, *p* < 0.001, suggesting consistent short-term reductions in negative affect within sessions (see Figure 2).

For PE, the model explained 67.2% of the variance when random effects were included (conditional R^2^ = 0.672), with 1.3% explained by fixed effects (marginal R^2^ = 0.013). Timepoint had a significant main effect, F(8, 523.4) = 2.38, *p* = 0.016, reflecting a small initial increase followed by a gradual decline over sessions. PE was significantly lower at the final session than at BL, t(31) = −1.98, *p* = 0.039. In contrast to NE, the pre–post condition effect was not significant, indicating no systematic within-session changes in PE (see Figure S1 in the Supplementary Material).

### 3.4. Course of Self-Esteem Height and Emotion Regulation Strategies

A linear mixed model for SEKJ-H across the seven available sessions showed no significant effect of timepoint, indicating that SE height remained stable over the course of the intervention (see Figure S2 in the Supplementary Material).

Pre–post comparisons of FEEL-KJ scores from BL to the final assessment revealed no statistically significant changes in total adaptive or maladaptive strategies, although there were trends towards increased humor enhancement and higher adaptive total scores and a trend towards reduced withdrawal. Among the “different” strategies, expression increased significantly, suggesting that adolescents reported more emotional expression at the end of the group. For more details, see Table S3 in the Supplementary Materials.

### 3.5. Exploratory Associations Between Affect, SE, and Clinical Characteristics

In line with the pilot character of the study, exploratory two-tailed Spearman correlations were calculated between affect (NE, PE), SE height (SEKJ-H), ER strategies (FEEL-KJ), problematic internet use (CIUS, AICA), temperament and character (JTCI), and psychopathology (YSR) across intervention time points. Full correlation matrices are provided in Tables S4–S6 in the Supplementary Material. In summary, higher adaptive ER and higher SE were generally associated with lower NE and higher PE, whereas higher maladaptive strategies and higher internalizing symptoms tended to correlate with higher NE and lower PE. Selected temperament and character traits (e.g., lower harm avoidance, higher self-directedness) showed favorable associations with affect and SE. Given the small sample size and lack of correction for multiple testing, these findings are exploratory and hypothesis-generating.

### 3.6. Drop out Analysis

Overall, no significant differences were observed between participants who completed all sessions and those who did not with respect to diagnostic burden, comorbidity, PIU, self-esteem, or internalizing and externalizing symptoms. Adaptive coping strategies assessed at baseline differed at a trend level between completers and dropouts (Mann–Whitney U = 225.5, z = 1.94, *p* = 0.052, r = 0.31). Participants who did not complete the intervention reported lower levels of adaptive strategies (Median = 104) compared with those who completed all sessions (Median = 125). No significant group differences were observed for self-esteem, maladaptive coping strategies, or internalizing and externalizing symptoms.

## 4. Discussion

The aim of this uncontrolled pilot and feasibility study was to evaluate an outpatient DBT-K–based skills training group on PE and NE, SE, and coping strategies in a transdiagnostic sample of adolescents with different temperaments, character traits, and heterogeneous psychopathological profiles. In line with this aim, we focused on short-term changes in affect, SE height, and ER strategies across eight group sessions, and on exploratory associations with PIU, temperament, and symptom severity.

In general, this intervention format—a creative adaptation of the DBT-A manual—was designed to offer an alternative to traditional, highly cognitive and worksheet-based skills training. The therapeutic setting explicitly aimed to avoid a “school-like” atmosphere and to create an easily accessible, engaging environment for adolescents. Playful elements (e.g., Origami) and expressive techniques (e.g., role play, storytelling) were implemented to enhance multimodal learning, which may facilitate internalization of content [54,55]. Despite an emphasis on adherence, participation was not mandatory in order to preserve the participants’ intrinsic motivation to attend sessions. The resulting dropout rate of 53% by session 8 is comparable to, or slightly lower than, the attrition rate reported in some DBT-A-based interventions (dropout rate approx. 60%) [56], but higher than in other adolescent DBT programs (dropout rate approx. 25%) [30,43]. In the only transdiagnostic DBT skills trial available to date, Neacsiu et al. [5] observed a dropout rate of 32% in their transdiagnostic DBT skills training group and a notably higher rate of 59% in their activities-based support group. Our rate of 53% falls within this range, suggesting that while DBT-K is implementable and acceptable in routine outpatient care, engagement and retention remain critical feasibility challenges for transdiagnostic interventions in highly burdened samples. Analyses comparing participants who dropped out and those who completed the intervention showed no significant differences in psychopathology, self-esteem, pathological Internet use, or psychiatric symptom burden. This suggests that dropout was not diagnosis-specific, thereby supporting the transdiagnostic scope of the intervention.

In line with the transdiagnostic focus, inclusion and exclusion criteria were intentionally broad. Most patients met criteria for anxiety-related disorders (n = 27), followed by personality disorders (n = 25) and mood/affective disorders (n = 7), and participants frequently met criteria for at least one additional disorder. This high comorbidity reflects the clinical complexity of the sample and supports the rationale for targeting shared mechanisms such as ER and SE rather than disorder-specific syndromes.

### 4.1. Main Findings: Changes in Affect

To examine whether NE decreased, PE increased, and SE height improved over the course of the intervention, linear mixed models were conducted. For NE, a significant reduction was observed when comparing BL to the final assessment. NE decreased significantly during the early sessions (from BL to session 4), followed by a small, temporary increase between sessions 5 and 6. Notably, ER content was the focus of sessions 4 and 5. A short-term increase in self-reported NE during this period does not necessarily indicate adverse outcomes. Instead, an apparent upward trend in negative affect may reflect increased awareness and more differentiated articulation of (negative) emotional states, rather than a true deterioration in mood. Another possible explanation could be the experience of internal conflicts between pre-existing emotional coping patterns and newly introduced adaptive strategies. Adolescents may have learned to avoid or suppress aversive emotions prior to treatment; when they begin to conceptualize negative emotions as normal and tolerable, they may be more likely to observe and report them.

After the ER modules, the most pronounced decrease in NE occurred between sessions 6 and 7. This pattern aligns with improved emotional well-being and may reflect the implementation of newly learned emotion regulation strategies. While DBT is typically offered to patients with borderline personality disorder in specialized facilities [28,34], the use of DBT-based techniques for a diagnostically heterogeneous outpatient population represents a novel approach. This underscores the value of transdiagnostic interventions and suggests that such techniques could be generalized to provide immediate support to patients in acute emotional distress. Given that emotion dysregulation and low self-esteem are core features across multiple psychopathologies [11,57], directly targeting these transdiagnostic processes constitutes a meaningful and increasingly relevant treatment strategy in modern psychotherapy [5,35]. The consistency within session reductions in NE observed across all eight modules further suggests that the creative, experiential nature of DBT-K offers outpatients rapid relief from emotional distress. Clinically, this indicates that brief creative group formats may serve as low-threshold stabilization tools in outpatient care, potentially reducing the need for more intensive emergency interventions during ongoing treatment. At the final assessment, NE exhibited a small and non-significant increase. This may reflect distress or anticipatory anxiety associated with the ending of a group-based, therapeutically supportive setting. Adolescents may experience attachment or transition-related anxiety at treatment termination [58], which could represent a normal emotional response rather than an indication of insufficient treatment effects.

Correlational analyses further showed that NE levels were negatively associated with BL adaptive strategies and positively associated with BL maladaptive strategies, as well as with internalizing symptoms during early to mid-sessions. This pattern is consistent with existing evidence for associations between maladaptive ER, internalizing symptoms, and negative affect [11,12]. In later sessions, NE remained negatively associated with adaptive strategies, whereas correlations with maladaptive strategies and internalizing symptoms weakened. Although no causal conclusions can be drawn, this attenuation could be consistent with the notion that repeated practice of skills may facilitate a partial decoupling of negative affect from maladaptive coping and symptom severity.

For PE, we observed a different pattern. After an initial minor increase from BL to session 1, PE gradually declined over subsequent sessions, which appears to contradict previous findings from DBT-based treatments emphasizing increases in PE [27]. Correlations between PE and adaptive strategies indicated that adolescents who used more functional strategies, such as cognitive reappraisal, tended to report higher levels of PE, which is aligned with prior research [59]. However, PE was positively (though not significantly) associated with externalizing problems, which does not fully fit the view by Aldao et al. [13] that successful ER protects against the development of externalizing disorders. Moreover, PE showed no significant association with adaptive strategies at sessions 4 and 5, when ER was explicitly addressed, diverging from theoretical and empirical work that links adaptive strategies to higher PE [11,60,61].

One possible interpretation could be that the brief intervention period and the high burden of ongoing stressors (e.g., school demands, family conflicts) limited the extent to which PE could increase, even if adolescents became somewhat better at managing negative affect. Additionally, enhancing PE may require more explicit and sustained focus on building pleasant activities, mastery experiences, and social reward (e.g., behavioral activation–type modules), beyond what was included in DBT-K [62,63]. It is also important to note that our sample included a high proportion of adolescents with internalizing disorders, primarily depression and anxiety. In these conditions, evidence-based psychotherapies typically show stronger effects in reducing negative affect than in increasing positive affect. Clinical trials and secondary analyses indicate that cognitive-behavioral interventions reliably improve depressive mood and distress, whereas gains in positive affect and reductions of anhedonia are more modest and often remain incomplete at follow-up, with anhedonia representing a common residual symptom [64,65]. A similar pattern has been reported for anxiety-related disorders, where psychotherapeutic interventions do not normalize positive and negative affect to comparable degrees [66]. The observed decrease in positive affect may also relate to patients’ expectations regarding therapeutic improvement. Expectancy effects have been shown to influence treatment outcomes in major depressive disorder, such that higher initial expectations of improvement are associated with greater symptom change, and mismatches between expected and actual progress can impact emotional experience and reward responsiveness. This mechanism may contribute to transiently reduced positive affect even in the context of overall symptom reduction [67].

For both NE and PE, the relatively large random-effect variance in the mixed models suggests substantial interindividual differences in trajectories, likely reflecting heterogeneity in psychopathology, life circumstances, and concurrent treatments rather than uniform intervention effects at the group level.

### 4.2. Self-Esteem, Emotion Regulation, and Problematic Internet Use

SE height remained relatively stable across all sessions, with fluctuations that did not reach statistical significance. The most noticeable increase was observed in session 6, which coincided with the SE/DO module, but even this change was small. The overall stability of SE contrasts with Rahmanzadeh et al. [42], who reported significant SE gains in a DBT-A intervention for male adolescents. This discrepancy raises a methodological question as to whether SE height is a sufficiently sensitive indicator of change in an eight-week group, given that SE is relatively stable during adolescence [68]. From this perspective, it could be argued that assessing components such as self-efficacy and self-respect—core elements of SE—might provide a more nuanced indicator of changes observed during the intervention period. As these facets were not measured in the present study, conclusions regarding SE as an outcome must remain tentative.

Despite limited change over time, SE height showed robust negative associations with NE and positive associations with PE across all intervention time points. These findings align with existing research on SE, ER, and coping, suggesting that adolescents with low SE are more prone to NE due to increased self-critical thinking, heightened sensitivity to failure, and lower perceived self-efficacy [69]. SE was also inversely related to maladaptive coping, internalizing and externalizing symptoms, supporting its role as a protective psychological resource against psychopathology [70,71].

A particularly notable feature of the sample was the high prevalence of problematic internet use (PIU). According to common cut-offs measured by CIUS [45,72], 28.3% (n = 15) of participants met subthreshold criteria for PIU, and 34% (n = 18) met full criteria. These rates are consistent with current research [73,74,75]. Adolescents with PIU tend to report lower SE, greater loneliness, and higher anxiety [24,76,77]. Low SE and unrealistic optimism have been identified as predictors of PIU, suggesting a bidirectional relationship between SE and problematic online behavior [78,79]. In the present study, however, we did not observe a clear correlational pattern between PIU and SE height across intervention sessions. Rather than contradicting previous findings, this likely reflects limited statistical power, measurement differences, and the complex, transdiagnostic nature of the sample. At the same time, the combination of high PIU and low SE underlines the potential benefit of integrating digital-media–specific modules into DBT-A–based programs. Such modules could explicitly address online behavior, digital boundaries, and ER in digital contexts.

Finally, we expected adaptive strategies to increase and maladaptive strategies to decrease over the course of the intervention. At BL, more than half of the adolescents scored below the test norm on adaptive strategies, whereas all participants scored within the norm range on maladaptive strategies. Pre-post assessments revealed only minimal normative change in adaptive coping (2% decrease in the proportion below norm). At the subscale level, trends towards increased humor enhancement and higher adaptive total scores emerged, and a trend towards decreased withdrawal was observed for maladaptive strategies. Since 77.4% of the sample exceeded the clinical cut-off point for internalizing symptoms and 7.5% were in the subthreshold range, even a small non-significant reduction in withdrawal, which is a core internalizing-related strategy [80,81], could be considered clinically relevant and suggest at least partial effectiveness. Additionally, a statistically significant change was observed in the expression subscale, indicating that adolescents more frequently expressed their emotions in the final assessment than in the initial one. This aligns with the central design feature of DBT-K, which provides a framework for adolescents to engage in creative and expressive activities throughout the group.

The significant increase in the expression subscale observed in our sample suggests that integrating creative and non-verbal modules is particularly beneficial for helping adolescents bridge the gap between emotional awareness and articulation. Furthermore, the high prevalence of problematic internet use (PIU) identified at baseline highlights a critical practical implication: clinicians should systematically screen for digital behavior in this population and consider integrating media-related coping strategies into standardized DBT-A-based protocols.

### 4.3. Limitations and Future Directions

The findings of this study need to be interpreted considering several important limitations. First, this was an uncontrolled, single-arm pilot study conducted in a naturalistic clinical setting. Without a control or comparison group, it is not possible to attribute observed changes in NE, PE, SE, or coping strategies specifically to the DBT-K intervention. Spontaneous improvement, regression to the mean, concurrent individual therapies, medication changes, or external factors (e.g., school or family events) may have contributed to the observed patterns. These methodological constraints suggest that the present findings should be interpreted as preliminary indicators of feasibility rather than conclusive evidence of clinical efficacy. Moreover, the extent to which these results generalize to other clinical settings without comparable concurrent support remains uncertain.

Second, the sample size was modest and predominantly female, which precluded gender-specific analyses and limited generalizability to boys and gender-diverse adolescents. Attendance declined over time, and many analyses, particularly those involving later sessions and final assessments, relied on reduced subsamples. Although linear mixed models are relatively robust to missing data under assumptions of missing at random, selective attrition (e.g., more severely impaired adolescents dropping out earlier) cannot be ruled out. Although no significant differences in psychopathology, comorbidity, internet use patterns, coping styles, or self-esteem were found between dropouts and completers, the factors contributing to dropout were not systematically assessed and thus remain unknown. As a result, our findings may overrepresent adolescents with higher motivation to engage in treatment or lower symptom severity, which restricts generalizability to individuals with more severe clinical presentations.

Third, the high dropout rate of 53% by session eight poses a significant challenge to interpreting the feasibility and generalizability of the intervention. This dropout rate is notably higher than the 25% typically reported in standard DBT-A programs [30,43]. However, it is consistent with other transdiagnostic pilot studies, for example, Neacsiu et al. reported a 32% dropout in their DBT skills training condition and a 59% dropout in their activities-based support group [5]. This suggests that maintaining engagement in a brief transdiagnostic format with a sample experiencing significant distress remains a systemic challenge. Unlike in structured research protocols, where additional treatments are kept constant, it has been shown that 45% of participants in similar transdiagnostic trials struggle with protocol compliance [5]. In our naturalistic setting, the lack of mandatory participation may have lowered the threshold for discontinuation. Furthermore, the developmental appropriateness of the creative content warrants critical reflection. Specific metaphors, such as the use of fairy tales (e.g., “Snow White” and “Sleeping Beauty”) in later sessions, could be perceived as patronizing or infantilizing by late adolescents (aged 16–18). Although these narratives provided a structured introduction to psychodrama, this potential mismatch between content and the participants’ developmental stage may have negatively impacted engagement, potentially contributing to the high attrition rate observed in later modules. As the reasons for dropping out were not systematically assessed, it is unclear whether the creative, less traditional format was perceived as insufficiently relevant or whether ongoing life stressors overwhelmed the participants.

Fourth, the intervention period was brief (approximately eight weeks), and no follow-up assessments were conducted. As a result, we were unable to examine the durability of changes in NE, expression, or other outcomes, or to detect potential delayed effects on more trait-like constructs such as SE and broader coping repertoires. Longer interventions and extended follow-up periods are likely necessary to capture more robust and sustained change [82].

Fifth, all measures relied on adolescent self-report, which can be influenced by response biases, current mood, insight, and social desirability. In a group setting, social desirability may be particularly relevant, as participants might overreport positive changes to align with perceived therapist expectations. Future studies would benefit from incorporating multiple informants (e.g., parents, teachers, clinicians) and additional methods such as behavioral tasks or ecological momentary assessment of affect and skill use [83].

Sixth, given the exploratory nature of this pilot study, a large number of correlational analyses were performed without correction for multiple testing. This approach is appropriate for hypothesis generation but increases the risk of type I error. The specific patterns of association observed between affect, SE, ER, PIU, and temperament should therefore be interpreted cautiously and replicated in larger samples with appropriate control for multiplicity [84].

Finally, we did not include direct DBT-specific process measures, such as daily skill use, perceived skill effectiveness, or diary card data [85]. This limits our ability to examine whether the creative group format effectively increased the use of DBT skills in everyday life and whether changes in skill use mediated the observed affective patterns.

Future research should retain the transdiagnostic design, as disorder-specific DBT-A studies remain underrepresented, but move towards larger, more diverse, and controlled trials. Randomized controlled designs comparing DBT-K to treatment-as-usual or alternative group programmes would allow for stronger conclusions about efficacy. Larger samples would also permit subgroup analyses (e.g., by gender, diagnostic cluster, PIU severity). Longer follow-up periods are needed to assess the sustainability of effects and potential delayed improvements in SE and coping [82]. In addition, future studies should consider implementing more sensitive measures of self-related constructs (e.g., self-efficacy, self-respect) while maintaining feasibility and engagement for adolescents [86]. Given the elevated rates of PIU in this sample, integrating digital or computer-based modules—for example, as blended or online components—may further enhance relevance and skills acquisition in digitally immersed youth.

## 5. Conclusions

With the rising prevalence of mental health disorders among children and adolescents, many young people continue to encounter substantial barriers to accessing timely and adequate treatment. The present creative, DBT-A-based skills group represents an exploratory attempt to address emotional and behavioral difficulties in a transdiagnostic outpatient setting. Within the constraints of an uncontrolled pilot design and modest sample size, participation in DBT-K was associated with reductions in negative emotions and small changes in emotion expression and coping strategies. However, the low levels of positive affect and self-esteem, combined with high attrition rates and a predominantly female sample, underscore that these preliminary findings should not be generalized to the broader adolescent psychiatric population.

The transdiagnostic framework nonetheless provides a useful conceptual lens for considering how future interventions may support adolescents with diverse clinical profiles. To determine whether such effects translate into sustainable clinical benefit, research employing controlled designs and more heterogeneous samples will be necessary. From a clinical standpoint, these early observations may offer a pragmatic orientation for addressing the high prevalence of emotion dysregulation and digital-media-related strain in routine outpatient care. Rather than relying exclusively on disorder-specific protocols, DBT-informed approaches may represent a flexible resource for targeting core transdiagnostic processes such as low self-esteem and affective instability across different adolescent presentations. In this sense, DBT-K may serve as one example of how feasibility, engagement, and mechanistic relevance could be balanced within future outpatient youth programs [5,37,61].

## Figures and Tables

**Table 1:** Distribution of psychopathological diagnoses according to the 10th revision of the International Statistical Classification of Diseases and Related Health Problems (ICD-10).

ICD-10 Category	Diagnostic Group	n (% ^1^)	Comorbidities ^2^ n (%)
F10–F19	Mental disorders due to psychoactive substance use	2 (3.8%)	-
F20–F29	Schizophrenia, schizotypal, delusional, and psychotic disorders	2 (3.8%)	-
F30–F39	Mood [affective] disorders	7 (13.2%)	3 (42.8%)
F40–F48	Anxiety, stress-related, somatoform disorders	27 (50.9%)	8 (29.6%)
F50–F59	Behavioral syndromes associated with physiological disturbances	4 (7.5%)	2 (50%)
F60–F69	Disorders of adult personality and behavior	25 (47.2%)	3 (12%)
F80–F89	Pervasive and specific developmental disorders	1 (1.9%)	-
F90–F98	Behavioral and emotional disorders with onset in childhood/adolescence	9 (17.0%)	2 (22.2%)

Note: ^1^ Percentages may sum up to more than 100% due to multiple diagnoses per patient (comorbidity). ^2^ Number of individuals with at least one additional psychiatric disorder, given that the primary diagnosis was the reason for admission.

## Data Availability

The data presented in this study are available on request from the corresponding author due to legal reasons in clinical populations.

## Supplementary Materials

The following supporting information can be downloaded at: https://www.mdpi.com/article/10.3390/children13020172/s1, Table S1. Descriptive statistics of baseline measures for age, internet use (AICA, CIUS), emotion regulation (FEEL-KJ), fear of missing out (FoMO), temperament and character (JTCI), affect (PANAS), self-esteem (SEKJ), and psychopathology (YSR); Table S2. Descriptive statistics of PANAS and SEKJ-H across the eight intervention time points and final assessment of PANAS, SEKJ, and FEEL-KJ; Table S3. Pairwise comparisons between FEEL-KJ baseline and final assessment (adaptive, maladaptive, and “other” strategies); Table S4. Two-tailed Spearman correlations between negative emotions (PANAS-NE) at all intervention time points and CIUS, FEEL-KJ (baseline and final), FoMO, JTCI, SEKJ, SEKJ-H, and YSR; Table S5. Two-tailed Spearman correlations between positive emotions (PANAS-PE) at all intervention time points and CIUS, FEEL-KJ (baseline and final), FoMO, JTCI, SEKJ, SEKJ-H, and YSR; Table S6. Two-tailed Spearman correlations between self-esteem height (SEKJ-H) across intervention time points and CIUS, AICA, FEEL-KJ (baseline and final), FoMO, JTCI, and YSR; Figure S1. Linear mixed model for positive emotions (PANAS-PE) across the intervention, including baseline; Figure S2. Linear mixed model for self-esteem height (SEKJ-H) across the intervention.

## Abbreviations

The following abbreviations are used in this manuscript:

AICA	Assessment of Internet and Computer Game Addiction
BL	Baseline
CIUS	Compulsive internet use scale
DBT	Dialectical behaviour therapy
DBT-A	Dialectical behaviour therapy for adolescents
DBT-K	Creative dialectical behaviour therapy for adolescents
DO	Dogmas
DT	Distress Tolerance
ER	Emotion regulation
FoMO	Fear of Missing Out
FEEL-KJ	Questionnaire for the evaluation of emotional regulation in children and adolescents (in German: Fragebogen zur Erhebung der Emotionsregulation bei Kindern und Jugendlichen)
ICD-10	10th revision of the International Statistical Classification of Diseases and Related Health Problems
IE	Interpersonal Effectiveness
JTCI	Junior Temperament and Character Inventory
MI	Mindfulness
NE	Negative emotions
PANAS	Positive and negative affect schedule
PE	Positive emotions
PIU	Problematic internet use
SE	Self-esteem
SEKJ	Self-esteem inventory for children and adolescents (in German: Selbstwertinventar für Kinder und Jugendliche)
SEKJ-H	Self-esteem inventory for children and adolescents height subscale
YSR	Youth Self-Report
