# Supplementary material for: DBT-K for Adolescents: Feasibility and Preliminary Outcomes of a Creative Eight-Week, DBT-Based Transdiagnostic Skills Group
Source: Children (Basel). 2026 Jan 26;13(2):172. doi: 10.3390/children13020172 (PMC12939821; doi:10.3390/children13020172)
Supplement: Supplementary file 1 [file children-13-00172-s001.zip › children-4087928-supplementary.pdf]

Supplementary Material: Table S1. Descriptive statistics of baseline measures for age, internet use (AICA, CIUS), emotion regulation (FEEL-KJ), fear of missing out (FoMO), temperament and character (JTCI), affect (PANAS), self-esteem (SEKJ) and psychopathology (YSR); Table S2. Descriptive statistics of PANAS and SEKJ-H across the eight intervention time points and final assessment of PANAS, SEKJ and FEEL-KJ; Table S3. Pairwise comparisons between FEEL-KJ baseline and final assessment (adaptive, maladaptive and “other” strategies); Table S4. Two-tailed Spearman correlations between negative emotions (PANAS-NE) at all intervention time points and CIUS, FEEL-KJ (baseline and final), FoMO, JTCI, SEKJ, SEKJ-H and YSR; Table S5. Two-tailed Spearman correlations between positive emotions (PANAS-PE) at all intervention time points and CIUS, FEEL-KJ (baseline and final), FoMO, JTCI, SEKJ, SEKJ-H and YSR; Table S6. Two-tailed Spearman correlations between self-esteem height (SEKJ-H) across intervention time points and CIUS, AICA, FEEL-KJ (baseline and final), FoMO, JTCI and YSR; Figure S1. Linear mixed model for positive emotions (PANAS-PE) across the intervention including baseline; Figure S2. Linear mixed model for self-esteem height (SEKJ-H) across the intervention

Table S1

Descriptive statistics of baseline measures for age, internet use measures assessed by the Assessment of Internet and Computer Game Addiction (AICA) and the Compulsive Internet Use Scale (CIUS), emotion regulation measured by the Questionnaire for the evaluation of emotional regulation in children and adolescents (FEEL-KJ), Fear of Missing Out (FoMO), affect assessed by the Positive and Negative Affect Scale (PANAS), temperament and character assessed by the Junior Temperament and Character Inventory (JTCI), self-esteem assessed by the Self-esteem Inventory for Children and Adolescents (SEKJ), and psychopathology assessed by the Youth Self-Report (YSR)

**Demographics**	*N*	*M*	*SD*	*Min*	*Max*
Age	53	15.6	1.4	13	19
**AICA**					
AICA CP score	53	2.2	3.4	0	14
AICA SNS score	53	7.0	6.3	0	23
AICA SM score	53	7.4	6.0	0	23
AICA Stream score	53	0.1	0.3	0	1
**CIUS**					
CIUS score	53	25.4	10.5	5	51
**FEEL-KJ (Baseline)**					
**Adaptive strategies**					
Problem solving	53	15.7	4.4	8	29
Within norm (%)	20 (37.7%)				
Below norm (%)	32 (60.3%)				

Distraction	53	13.4	5.6	6	27
Within norm (%)	17 (32.1%)				
Below norm (%)	43 (81.1%)				
Humor Enhancement	53	12.1	5.1	6	27
Within norm (%)	15 (28,3%)				
Below norm (%)	37 (69.1%)				
Acceptance	53	14.0	4.7	6	25
Within norm (%)	2 (3.8%)				
Below norm (%)	30 (56.6%)				
Forgetting	53	16.0	4.5	8	25
Within norm (%)	28 (52.8%)				
Below norm (%)	24 (45.3%)				
Cognitive Problem Solving	53	19.0	5.7	6	29
Within norm (%)	26 (49.1%)				
Below norm (%)	20 (37.7%)				
Revaluation	53	16.0	6.3	6	30
Within norm (%)	15 (28.3%)				
Below norm (%)	26 (49.1%)				
Total	53	106.2	26.5	52	166
Within norm (%)	22 (41.5%)				
Below norm (%)	30 (56.6%)				
**Maladaptive strategies**					
Giving Up	53	22.0	5.0	9	30
Within norm (%)	14 (26.4%)				
Below norm (%)	2 (3.8%)				
Aggression	53	12.6	5,9	6	29
Within norm (%)	30 (56.6%)				
Below norm (%)	8 (15.0%)				
Withdrawal	53	24.0	5.0	8	30
Within norm (%)	12 (22.6%)				
Below norm (%)	1 (1.8%)				
Self-Devaluation	53	24.6	4.8	11	30
Within norm (%)	13 (24.5%)				
Below norm (%)	1 (1.9%)				
Perseveration	53	23.2	4.6	12	30

Within norm (%)	25 (47.1%)				
Below norm (%)	2 (3.8%)				
Total	53	104.8	19.3	17	134
Within norm (%)	53 (100%)				
Below norm (%)	-				
**Different strategies**					
Social Support	53	15.6	6.2	6	30
Within norm (%)	30 (56.6%)				
Below norm (%)	20 (37.7%)				
Expression	53	15.4	5.6	6	30
Within norm (%)	33 (62.2%)				
Below norm (%)	13 (24.5%)				
Emotion Control	53	21.6	5.0	8	30
Within norm (%)	21 (39.6%)				
Below norm (%)	2 (3.8%)				
**FoMO Scale**					
FoMO score	53	29.3	8.9	14	50
**JTCI Scale**					
Novelty Seeking score	52	28.3	11.6	1	49
Within norm (%)	32 (61.5%)				
Below norm (%)	12 (23.1%)				
Harm avoidance score	52	40.4	7.4	21	59
Within norm (%)	7 (13.4%)				
Below norm (%)	-				
Reward dependence score	52	35.0	11.3	13	59
Within norm (%)	27 (51.9%)				
Below norm (%)	18 (34.6%)				
Persistence score	52	25.9	9.6	9	47
Within norm (%)	29 (55.8%)				
Below norm (%)	21 (40.4%)				
Self-guidance score	52	19.9	9.8	24	66
Within norm (%)	8 (15.4%)				
Below norm (%)	44 (84.6%)				
Cooperativity score	52	50.2	8.7	24	66
Within norm (%)	36 (69.2%)				

Below norm (%)	5 (9.6%)				
Self-transcendence score	52	17.9	6.9	2	38
Within norm (%)	40 (76.9%)				
Below norm (%)	4 (7.6%)				
**PANAS Baseline**					
Positive Emotion	33	13.4	8.8	1	38
Negative Emotion	33	15.4	9.1	1	36
**SEKJ Baseline**					
Self-esteem Height	53	21.0	8.4	10	43
Within norm (%)	8 (15.1%)				
Below norm (%)	45 (84.9%)				
Self-esteem Stability	53	20.8	7.9	11	40
Within norm (%)	18 (34%)				
Below norm (%)	35 (66%)				
Self-esteem Contingency	53	27.0	11.5	12	53
Within norm (%)	16 (30.2%)				
Below norm (%)	36 (67.9%)				
**YSR**					
Internalizing symptoms	53	36.5	15.1	9	113
Within norm (%)	3 (5.6%)				
Below norm (%)	-				
Externalizing symptoms	53	18.9	10.4	2	44
Within norm (%)	22 (41.5%)				
Below norm (%)	-				

Note: N = Number of participants, M = Mean, SD = Standard Deviation, Min = Minimum Score, Max = Maximum Score. AICA = Assessment of Internet and Computer Game Addiction, CIUS = Compulsive Internet Use Scale, FEEL-KJ = Questionnaire for the evaluation of emotional regulation in children and adolescents, FoMO = Fear of Missing Out, PANAS = Positive and Negative Affect Scale, JTCI = Junior Temperament and Character Inventory, SEKJ = Self-esteem Inventory for Children and Adolescents, YSR= Youth Self-Report. Percentages are rounded and may differ from exact values.

Table S2

Descriptive statistics of Positive and Negative Affect Scale (PANAS) and Self-esteem Inventory for Children and Adolescents height (SEKJ-H) of eight intervention timepoints and final assessment of PANAS, SEKJ and the Questionnaire for the evaluation of emotional regulation in children and adolescents (FEEL-KJ).

Session 1	*N*	*M*	*SD*	*Min*	*Max*
**PANAS Pre Intervention**					
Positive Emotion	34	12.7	8.9	1	38
Negative Emotion	34	13.9	8.2	0	30
**PANAS Post Intervention**					
Positive Emotion	36	13.3	8.7	1	32
Negative Emotion	36	7.0	5.5	0	22
**SEKJ**					
Self-esteem Height	22	22.3	8.0	11	40
Within norm (%)	16 (72.7%)				
Below norm (%)	6 (27.3%)				
**Session 2**					
**PANAS Pre Intervention**					
Positive Emotion	41	10.7	8.3	0	36
Negative Emotion	41	10.6	8.1	1	31
**PANAS Post Intervention**					
Positive Emotion	41	10.8	9.9	0	38
Negative Emotion	41	7.8	7.1	0	32
**SEKJ**					
Self-esteem Height	41	22.3	8.0	11	40
Within norm (%)	9 (21.9%)				
Below norm (%)	32 (78.1%)				
**Session 3**					
**PANAS Pre Intervention**					
Positive Emotion	40	11.7	9.9	1	38
Negative Emotion	40	9.4	7.1	0	27
**PANAS Post Intervention**					
Positive Emotion	40	12.6	9.6	0	35
Negative Emotion	40	7.4	6.4	0	25
**SEKJ**					
Self-esteem Height	43	23.6	8.7	10	40
Within norm (%)	40 (93%)				
Below norm (%)	3 (7%)				
**Session 4**					
**PANAS Pre Intervention**					

Positive Emotion	39	9.8	8.4	0	34
Negative Emotion	39	10.3	6.5	0	30
**PANAS Post Intervention**					
Positive Emotion	40	11.1	9.5	1	37
Negative Emotion	40	6.7	6.5	0	25
**SEKJ**					
Self-esteem Height	36	21.1	9.0	0	41
Within norm (%)	9 (25%)				
Below norm (%)	27 (75%)				
**Session 5**					
**PANAS Pre Intervention**					
Positive Emotion	39	8.9	7.9	0	36
Negative Emotion	39	9.9	6.8	0	27
**PANAS Post Intervention**					
Positive Emotion	39	10.1	8.5	0	36
Negative Emotion	39	7.9	7.2	0	27
**SEKJ**					
Self-esteem Height	38	21.6	9.2	0	41
Within norm (%)	12 (31.6%)				
Below norm (%)	26 (68.4%)				
**Session 6**					
**PANAS Pre Intervention**					
Positive Emotion	34	9.9	10.0	0	37
Negative Emotion	34	10.2	8.2	0	29
**PANAS Post Intervention**					
Positive Emotion	34	10.9	10.4	0	35
Negative Emotion	34	8.6	7.8	0	28
**SEKJ**					
Self-esteem Height	33	22.7	9.6	10	42
Within norm (%)	10 (30.3%)				
Below norm (%)	23 (69.7%)				
**Session 7**					
**PANAS Pre Intervention**					
Positive Emotion	32	10.2	8.52	0	30
Negative Emotion	33	8.2	6.7	0	26

**PANAS Post Intervention**					
Positive Emotion	32	10.3	9.8	0	33
Negative Emotion	32	6.4	6.5	0	28
**SEKJ**					
Self-esteem Height	32	22.3	9.1	10	42
Within norm (%)	7 (21.9%)				
Below norm (%)	25 (78.1%)				
**Session 8 (Final Assessment)**					
**PANAS Pre Intervention**					
Positive Emotion	28	9.7	9.97	0	38
Negative Emotion	28	11.0	8.3	0	29
**PANAS Post Intervention**					
Positive Emotion	28	10.5	10.6	0	39
Negative Emotion	28	8.4	8.2	0	33
**SEKJ**					
Self-esteem Height	29	37.1	12.0	21	75
Within norm (%)	7 (24.1%)				
Below norm (%)	21 (72.4%)				
Self-esteem Stability	29	35.6	12.4	21	67
Within norm (%)	5 (17.2%)				
Below norm (%)	22 (75.9%)				
Self-esteem Contingency	29	40.1	11.5	23	71
Within norm (%)	11 (37.9%)				
Below norm (%)	17 (58.6%)				
**FEEL-KJ**					
**Adaptive strategies**					
Problem solving	29	15.6	5.6	6	30
Within norm (%)	9 (16.9%)				
Below norm (%)	18 (33.9%)				
Distraction	29	13.8	5.6	6	26
Within norm (%)	12 (22.6%)				
Below norm (%)	17 (32.1%)				
Humor Enhancement	29	13.2	5.8	6	28
Within norm (%)	13 (24.5%)				
Below norm (%)	15 (28.3%)				

Acceptance	29	14.6	5.4	6	25
Within norm (%)	15 (28.3%)				
Below norm (%)	12 (22.6%)				
Forgetting	29	16.7	5.4	6	25
Within norm (%)	17 (32.1%)				
Below norm (%)	11 (20.7%)				
Cognitive Problem Solving	29	18.1	5.7	6	30
Within norm (%)	13 (24.5%)				
Below norm (%)	13 (24.5%)				
Revaluation	29	17.0	5.3	8	29
Within norm (%)	14 (26.4%)				
Below norm (%)	8 (15.1%)				
Total	29	109.0	30.6	44	172
Within norm (%)	14 (48.3%)				
Below norm (%)	14 (48.3%)				
**Maladaptive strategies**					
Giving Up	29	22.9	5.6	10	30
Within norm (%)	7 (24.1%)				
Below norm (%)	1 (3.4%)				
Aggression	29	12.9	5.7	6	29
Within norm (%)	14 (48.3%)				
Below norm (%)	4 (13.8%)				
Withdrawal	29	23.8	5.1	13	30
Within norm (%)	8 (27.6%)				
Below norm (%)	-				
Self-Devaluation	29	24.1	4.9	14	30
Within norm (%)	9 (31%)				
Below norm (%)	-				
Perseveration	29	23.4	4.9	13	30
Within norm (%)	12 (41.4%)				
Below norm (%)	2 (6.7%)				
Total	29	107.2	17.0	73	149
Within norm (%)	4 (13.8%)				
Below norm (%)	-				
**Different strategies**					

Social Support	29	15.8	5.5	6	29
Within norm (%)	18 (62.1%)				
Below norm (%)	10 (34.5%)				
Expression	29	16.5	4.2	9	26
Within norm (%)	22 (75.9%)				
Below norm (%)	4 (18.2%)				
Emotion Control	29	21.0	3.8	10	27
Within norm (%)	17 (58.6%)				
Below norm (%)	1 (3.4%)				

Note: N = Number of participants, M = Mean, SD = Standard Deviation, Min = Minimum Score, Max = Maximum Score. PANAS = Positive and Negative Affect Scale, FEEL-KJ = Questionnaire for the evaluation of emotional regulation in children and adolescents, SEKJ = Self-esteem Inventory for Children and Adolescents. Percentages are rounded and may differ from exact values.

Table S3

Pairwise comparisons between the Questionnaire for the evaluation of emotional regulation in children and adolescents (FEEL-KJ) at baseline and at the final assessment.

	Baseline		Final		Test	*p*	Effect size
	M	*SD*	*M*	*SD*			
**Adaptive strategies**							
Problem solving	15.34	4.8	15.59	5.6	-0.37	.357	-0.07
Distraction	13.03	5.6	13.83	5.6	177.0[a]	.560	-0.13[b]
Humor Enhancement	11.66	5.2	13.17	5.8	-1.49	.074	-0.27
Acceptance	13.59	4.4	14.62	5.4	-1.09	.144	-0.21
Forgetting	15.93	4.7	16.69	5.4	-0.72	.238	-0.13
Cognitive Problem Solving	18.72	4.6	18.07	5.7	0.97	.830	0.18
Revaluation	15.38	5.2	17.03	6.3	-1.30	.102	-0.24
Total	103.59	23.9	109.00	30.6	145.5[a]	.061	-0.33[b]
**Maladaptive strategies**							
Giving Up	21.93	5.8	22.86	5.6	-0.97	.831	-0.18
Aggression	11.79	5.4	12.97	5.7	174.0[a]	.751	-0.14[b]
Withdrawal	24.66	5.6	23.83	5.1	231.5[a]	.079	0.32[b]
Self-Devaluation	23.69	5.1	24.14	4.7	-0.59	.720	-0.11
Perseveration	23.48	4.7	23.41	5.0	0.09	.463	0.02
Total	102.83	23.5	107.21	17.0	167.0[a]	.797	-0.17[b]

**Different strategies**							
Social Support	15.97	5.7	15.79	5.5	0.18	.571	0.03
Expression	15.21	5.6	16.48	4.2	-1.79	**.042***	-0.33
Emotion Control	22.10	4.7	21.00	3.8	1.40	.173	0.26

Note: Based on n = 29 observations. [a] Wilcoxon W due to violation of the assumption of normality (Shapiro Wilk). [b] Effect size is reported as Cohen's d or point-biserial correlation if Wilcoxon W was used. Significant results are marked bold.
* Significance level at p < .05. ** Significance level at p < .01. *** Significance level at p < .001.

Table S4

Two tailed Spearman's correlations between negative emotions (PE) measured by the Positive and Negative Affect Scale (PANAS) across all intervention timepoints Compulsive Internet Use Scale (CIUS), baseline and final scores of the Questionnaire for the evaluation of emotional regulation in children and adolescents (FEEL-KJ), Fear of Missing Out (FoMO), Junior Temperament and Character Inventory (JTCI), baseline self-esteem and self-esteem intervention scores measured by Self-esteem Inventory for Children and Adolescents (SEKJ) and Youth Self-Report (YSR)

				PANAS				
Variables	NE1	NE2	NE3	NE4	NE5	NE6	NE7	NE8
CIUS	.08	.16	.15	.11	.00	.14	.04	-.15
FEEL-KJ AD (BL)	**-.52*****	**-.50****	-.26	-.28	**-.39***	**-.38***	-.19	-.09
FEEL-KJ MD (BL)	**.62*****	**.47*****	.27	.22	**.32***	.08	.04	.09
FEEL-KJ AD (final)	-.13	-.20	-.09	-.22	**-.40***	**-.61****	-.14	-.23
FEEL-KJ MD (final)	.33	**.45***	.13	.04	.30	.25	.07	.34
FoMO	.07	.01	.20	.01	-.18	-.15	-.13	-.20
JTCI Novelity seeking	.12	-.04	.13	-.24	-.14	.09	.19	.09
JTCI Harm avoidance	.32	.32	.25	.33	.30	.34	.07	-.19
JTCI Reward dependance	.10	-.09	.09	-.07	-.13	-.02	.20	.08
JTCI Persistence	.01	-.17	-.19	-.22	-.26	-.14	.04	.01
JTCI Self guidance	-.22	-.24	-.21	-.23	-.23	-.26	-.02	-.31
JTCI Cooperativity	-.22	-.19	-.26	-.26	**-.40***	-.19	-.23	.00
JTCI Self transcendence	-.02	-.17	-.07	**-.35***	-.09	-.06	.02	-.09
SEKJ-Height (BL)	-.31	-.30	-.21	-.26	**-.44****	-.31	-.07	-.22
SEKJ-Stability (BL)	-.16	.02	.18	.06	.10	-.17	-.22	.13
SEKJ-Contingency (BL)	-.19	-.05	-.08	**-.37***	-.13	-.13	-.18	**.46***
SEKJ-H T1	-.09	.09	-.04	.00	**-.57*****	-.38	-.12	.12
SEKJ-H T2	-.29	**-.46****	-.23	-.21	**-.44***	-.42*	-.16	-.33

SEKJ-H T3	-.25	**-.35***	-.20	-.34	-.27	**-.44***	-.22	-.08
SEKJ-H T4	-.19	-.20	-.17	-.25	-.21	.06	-.02	.13
SEKJ-H T5	**-.47***	-.26	-.20	-.24	**-.57*****	-.23	-.03	-.26
SEKJ-H T6	**-.41***	**-.38***	-.07	-.19	-.35	**-.39***	-.29	-.42
SEKJ-H T7	-.34	**-.47***	-.14	-.20	-.26	-.26	-.22	-.16
YSR Internalizing symptoms	.31	**.41****	.27	**.32***	**.40***	.33	-.04	.09
YSR Externalizing symptoms	**.34***	.16	.22	.10	.13	.20	.29	.07

Note: PANAS NE1-8 = Negative emotions assed by the Positive and Negative Affect Scale of intervention timepoint 1-8, CIUS = Compulsive Internet Use Scale, FEEL-KJ AD = Adaptive Strategies Subscale Scores assessed by the Questionnaire for the evaluation of emotional regulation in children and adolescents, FEEL-KJ MD = Maladaptive Strategies Subscale Scores assessed by the Questionnaire for the evaluation of emotional regulation in children and adolescents, BL= at baseline, final = final/last assessment timepoint, FoMO = Fear of Missing Out, JTCI = Junior Temperament and Character Inventory, SEKJ = Self-esteem Inventory for Children and Adolescents, SEKJ-H T1-7 = Self-esteem height assessed by the Self-esteem Inventory for Children and Adolescents of intervention timepoint 1-7, YSR = Youth Self-Report. Significant results are marked bold.
* Significance level at p < .05. ** Significance level at p < .01. *** Significance level at p < .001.

Table S5

Two tailed Spearman's correlations between positive emotions (PE) measured by the Positive and Negative Affect Scale (PANAS) across all intervention timepoints Compulsive Internet Use Scale (CIUS), baseline and final scores of the Questionnaire for the evaluation of emotional regulation in children and adolescents (FEEL-KJ), Fear of Missing Out (FoMO), Junior Temperament and Character Inventory (JTCI), baseline self-esteem and self-esteem intervention scores measured by Self-esteem Inventory for Children and Adolescents (SEKJ) and Youth Self-Report (YSR)

					PANAS			
Variables	PE1	PE2	PE3	PE4	PE5	PE6	PE7	PE8
CIUS	-.03	-.12	-.20	-.09	-.01	.03	-.24	-.04
FEEL-KJ AD (BL)	.32	.15	**.41****	**.39***	**.40***	.34	.23	.22
FEEL-KJ MD (BL)	.03	-.14	-.04	-.18	-.15	.02	-.18	.05
FEEL-KJ AD final	**.37***	**.44***	**.46***	.31	.33	**.64*****	**.65*****	**.53****
FEEL-KJ MD final	-.05	-.27	-.10	-.11	**-.41***	-.24	**-.41***	-.18
FOMO	.13	.04	.20	.07	.12	.20	.08	.20
JTCI Novelty seeking	.22	.16	.12	**.47****	.17	**.42***	**.52****	.40
JTCI Harm avoidance	-.29	**-.36***	-.25	**-.33***	-.25	-.20	-.24	-.31
JTCI Reward dependance	.31	.26	.12	.18	.15	.13	.19	**.58*****

JTCI Persistence	.18	.25	.33*	.08	.19	.36*	.34	**.42***
JTCI Self guidance	.39*	.31	.24	.25	.36*	.15	.296	**.39***
JTCI Cooperativity	.09	.14	.42**	.12	.27	.28	.20	.33
JTCI Self transcendence	.03	.11	-.18	.27	.12	.16	.07	.25
SEKJ-Height (BL)	**.37***	**.46****	.29	**.40***	**.63*****	**.39***	.32	**.40***
SEKJ-Stability (BL)	.15	-.04	.11	-.02	-.07	-.03	-.10	.06
SEKJ-Contingency (BL)	-.01	.17	.19	**.33***	.02	.22	.25	.21
SEKJ-H T1	.38	**.56****	-.14	.25	**.54***	.398	-.06	.44
SEKJ-H T2	**.37***	**.52*****	.34	.30	**.62*****	**.50***	.36	**.45***
SEKJ-H T3	.28	.18	**.49*****	.33	**.38***	.34	.26	.35
SEKJ-H T4	.27	.32	.28	**.38***	**.38***	.23	.26	.33
SEKJ-H T5	**.46***	**.45****	.21	**.41***	**.50*****	**.49****	.197	**.43***
SEKJ-H T6	**.52***	**.45***	**.50***	.25	.52**	**.59*****	**.58*****	**.63*****
SEKJ-H T7	.24	.17	.32	.23	.33	**.57*****	**.55*****	**.43***
YSR Internalizing symptoms	-.26	**-.42****	-.28	-.18	-.21	-.13	-.14	-.28
YSR Externalizing symptoms	.12	.08	-.01	.27	.07	.22	.26	.28

Note: PANAS PE1-8 = Positive emotions assed by the Positive and Negative Affect Scale of intervention timepoint 1-8, CIUS = Compulsive Internet Use Scale, FEEL-KJ AD = Adaptive Strategies Subscale Scores assessed by the Questionnaire for the evaluation of emotional regulation in children and adolescents, FEEL-KJ MD = Maladaptive Strategies Subscale Scores assessed by the Questionnaire for the evaluation of emotional regulation in children and adolescents, BL= at baseline, final = final/last assessment timepoint, FoMO = Fear of Missing Out, JTCI = Junior Temperament and Character Inventory, SEKJ = Self-esteem Inventory for Children and Adolescents, SEKJ-H T1-7 = Self-esteem height assessed by the Self-esteem Inventory for Children and Adolescents of intervention timepoint 1-7, YSR = Youth Self-Report. Significant results are marked bold.
* Significance level at p < .05. ** Significance level at p < .01. *** Significance level at p < .001.

Table S6

Two tailed Spearman's correlations between Self-esteem Inventory for Children and Adolescents (SEKJ) height subscales scores across seven intervention timepoints and Compulsive Internet Use Scale (CIUS), baseline and final scores of the Questionnaire for the evaluation of emotional regulation in children and adolescents (FEEL-KJ), Fear of Missing Out (FoMO), Junior Temperament and Character Inventory (JTCI), and Youth Self-Report (YSR)

Variables	SEKJ-H						
	SEKJ-H T1	SEKJ-H T2	SEKJ-H T3	SEKJ-H T4	SEKJ-H T5	SEKJ-H T6	SEKJ-H T7

CIUS	.31	-.23	-.03	-.07	.03	.03	-.15
AICA CP	-.03	-.15	.04	-.02	.13	.05	-.07
AICA SNS	.05	-.25	-.01	-.31	-.16	-.13	-.10
AICA SM	.25	-.23	-.01	-.20	-.05	-.05	.03
AICA Stream	-.07	.07	**.39***	.30	.10	.15	.28
FEEL-KJ AD (BL)	-.00	.26	**.33***	.09	.22	.33	.25
FEEL-KJ MD (BL)	.05	**-.41****	-.28	**-.51****	-.31	-.17	-.31
FEEL-KJ AD (final)	-.05	**.41***	**.41***	.23	.34	**.68****	**.56*****
FEEL-KJ MD (final)	.13	**-.51***	-.31	-.13	**-.42***	**-.52***	-.35
FoMO	-.19	-.16	.08	-.19	.02	.07	-.01
JTCI Novelity seeking	-.09	-.11	-.12	.07	.06	.03	.18
JTCI Harm avoidance	-.17	**-.44***	**-.34***	-.33	-.26	-.19	-.22
JTCI Reward dependance	-.06	.04	.10	-.06	.20	.17	-.00
JTCI Persistence	-.02	.31	**.34***	.32	.20	.30	**.46****
JTCI Self guidance	.22	**.61*****	**.46****	**.35***	**.44****	.46**	**.36****
JTCI Cooperativity	-.004	.03	.15	-.13	.20	.22	.05
JTCI Self transcendence	.18	.00	-.14	.15	.22	.02	.05
YSR Internalizing symptoms	-.30	**-.56*****	**-.34***	-.26	**-.37***	-.27	-.16
YSR Externalizing symptoms	-.28	-.25	-.28	-.18	-.15	-.09	-.06

Note: SEKJ-H T1-7 = Self-esteem height assessed by the Self-esteem Inventory for Children and Adolescents of intervention timepoint 1-7, CIUS = Compulsive Internet Use Scale, FEEL-KJ AD = Adaptive Strategies Subscale Scores assessed by the Questionnaire for the evaluation of emotional regulation in children and adolescents, FEEL-KJ MD = Maladaptive Strategies Subscale Scores assessed by the Questionnaire for the evaluation of emotional regulation in children and adolescents, BL= at baseline, final = final/last assessment timepoint, FoMO = Fear of Missing Out, JTCI = Junior Temperament and Character Inventory, YSR = Youth Self-Report. Significant results are marked bold. * Significance level at p < .05. ** Significance level at p < .01. *** Significance level at p < .001.

Figure S1

Linear Mixed Model for positive emotions scores measured by the Positive and Negative Affect Scale (PANAS) across the intervention (sessions) including baseline measure.

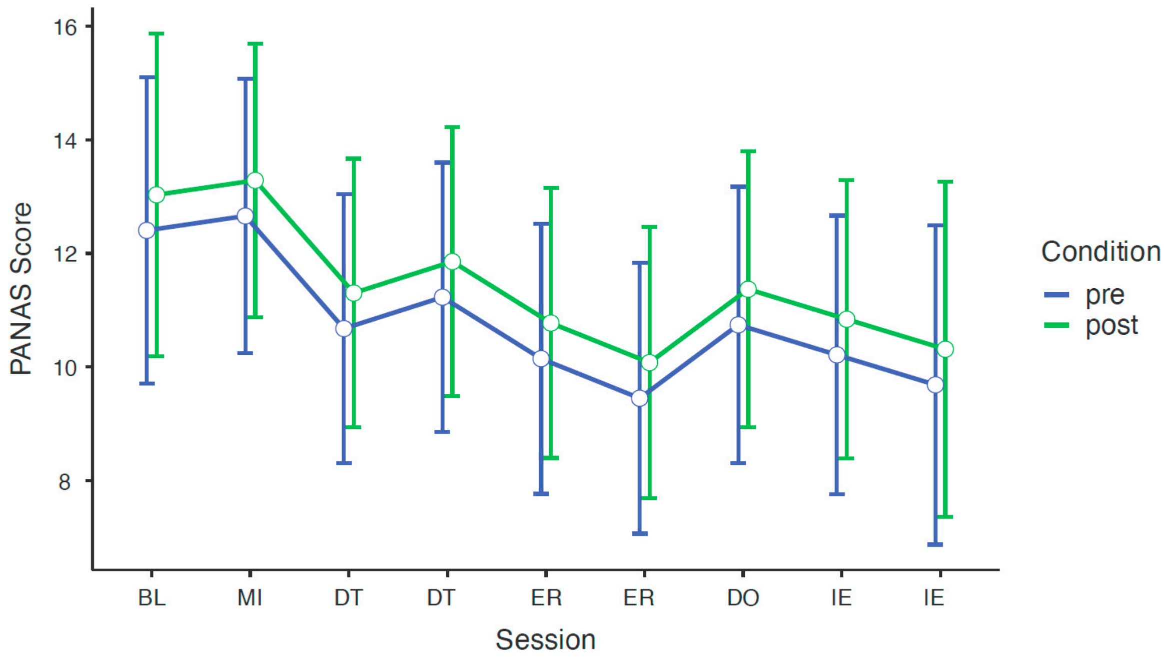

Note: PANAS = Positive and Negative Affect Scale, BL = Baseline, MI = Mindfulness, DT = Distress Tolerance, ER = Emotion Regulation, DO = Dogmas, IE = Interpersonal Effectiveness. Error bars represent 95% confidence interval. Pre = pre intervention, Post = post intervention.

Figure S2

Linear Mixed Model for self-esteem (SE) height scores measured by Self-esteem Inventory for Children and Adolescents across the intervention (sessions)

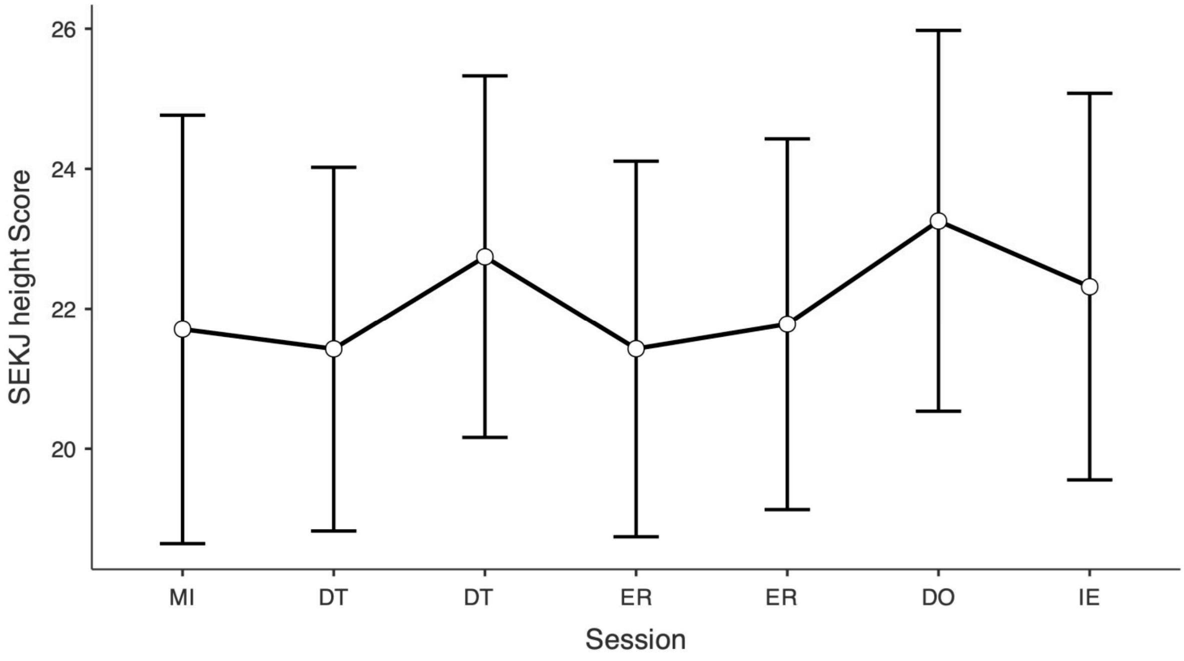

Note: MI = Mindfulness, DT = Distress Tolerance, ER = Emotion Regulation, DO = Dogmas, IE = Interpersonal Effectiveness. Error bars represent 95% confidence interval.
